# Characterization of cotton ARF factors and the role of GhARF2b in fiber development

**DOI:** 10.1186/s12864-021-07504-6

**Published:** 2021-03-22

**Authors:** Xiufang Zhang, Junfeng Cao, Chaochen Huang, Zishou Zheng, Xia Liu, Xiaoxia Shangguan, Lingjian Wang, Yugao Zhang, Zhiwen Chen

**Affiliations:** 1 National Key Laboratory of Plant Molecular Genetics and National Center for Plant Gene Research, Institute of Plant Physiology and Ecology/CAS Center for Excellence in Molecular Plant Sciences, Chinese Academy of Sciences, Shanghai, 200032 China; 2 Plant Stress Biology Center, Institute of Plant Physiology and Ecology/CAS Center for Excellence in Molecular Plant Sciences, Chinese Academy of Sciences, Shanghai, 200032 China; 3 University of Chinese Academy of Sciences, Shanghai, 200032 China; 4 School of Life Science and Technology, ShanghaiTech University, Shanghai, 201210 China; 5 Esquel Group, 25 Harbour Road, Wanchai, Hong Kong, China; 6 Institute of Carbon Materials Science, Shanxi Datong University, Datong, 037009 China

**Keywords:** Cotton, GhARF2b, Fiber elongation, Fiber initiation

## Abstract

**Background:**

Cotton fiber is a model system for studying plant cell development. At present, the functions of many transcription factors in cotton fiber development have been elucidated, however, the roles of auxin response factor (ARF) genes in cotton fiber development need be further explored.

**Results:**

Here, we identify auxin response factor (ARF) genes in three cotton species: the tetraploid upland cotton *G. hirsutum*, which has 73 ARF genes, and its putative extent parental diploids *G. arboreum* and *G. raimondii*, which have 36 and 35 ARFs, respectively. Ka and Ks analyses revealed that in *G. hirsutum ARF* genes have undergone asymmetric evolution in the two subgenomes. The cotton ARFs can be classified into four phylogenetic clades and are actively expressed in young tissues. We demonstrate that *GhARF2b*, a homolog of the Arabidopsis *AtARF2*, was preferentially expressed in developing ovules and fibers. Overexpression of *GhARF2b* by a fiber specific promoter inhibited fiber cell elongation but promoted initiation and, conversely, its downregulation by RNAi resulted in fewer but longer fiber. We show that GhARF2b directly interacts with GhHOX3 and represses the transcriptional activity of GhHOX3 on target genes.

**Conclusion:**

Our results uncover an important role of the ARF factor in modulating cotton fiber development at the early stage.

**Supplementary Information:**

The online version contains supplementary material available at 10.1186/s12864-021-07504-6.

## Background

Cotton is the most important natural and renewable material for the textile industry in the world [1]. The primary cultivated species upland cotton (*G. hirsutum* L.) is grown in over 80 countries and accounts for more than 90% of global cotton fiber output. Cotton fibers are unusually long, single-celled epidermal seed trichomes and a model for plant cell growth research [2]. Fiber development can be divided into four overlapping stages: initiation, elongation, secondary cell wall biosynthesis and maturation [3]. The fiber length and density are both key traits that determine cotton quality and yield.

The study of cotton fiber development regulation provides not only valuable knowledge to understanding plant cell growth and cell wall biosynthesis, but also candidate genes for cotton molecular breeding [4]. To date a number of genes that function in cotton fiber cells have been identified, including homeodomain transcription factor GaHOX1, GhHOX3 and GhHD1 [5–7], bHLH transcription factor GhPRE1 [8], KNOX transcription factor knl1 [9], the sterol carrier gene [10], MYB transcription factors GhMYB25, GhMYB25-like, GhMML3 and GhMML4 [11–14], NAC transcription factor fsn1 [15], transcription factor WLIM1a gene [16], sucrose synthase gene [17], cotton actin1 gene [18], cotton BURP domain protein GhRDL1 [19], ethylene pathway related genes [20], fasciclin-like arabinogalactan protein, Ghfla1 [21], and TCP transcription factor GhTCP4 [22] etc. Among recent progresses are the characterizations of transcription factors which regulate the major events of cotton fiber development, such as MYBs and HD-ZIP IVs involved in cotton fiber initiation and elongation, as well as a number of other types of factors. The MIXTA type MYB transcription factors (GhMYB25, GhMYB25-like and GhMML4_D12) are master regulators of cotton fiber initiation [11, 13, 14] and lint fiber development [12], whereas the HD-ZIP IV transcription factor GhHOX3 plays a pivotal role in controlling fiber elongation [5], whose activity is regulated by the phytohormone gibberellin. In addition, NAC (GhFSN1) and TCP4 transcription factors positively regulates secondary cell wall biosynthesis [15, 22]. However, cotton fiber growth and development are complex processes involving cell differentiation, cell skeleton orientation growth, cell wall synthesis, and so on [23]. Currently the picture of the regulation network of cotton fiber is far from complete.

Auxin response factors (ARFs), a group of plant transcription factors, are composed of a conserved N-terminal DNA binding domain (DBD), a most case conserved C-terminal dimerization domain (CTD) and a non-conserved middle region (MR) [24]. The MR region has been proposed to function as a repression or an activation domain [25]. *Arabidopsis thaliana* contains 23 ARF genes and *Oryza sativa* has 25 [26, 27]. It has been reported that ARF2 negatively modulates plant growth in *A. thaliana* [26, 28–30] and tomato [31], yet functions of transcription factors can vary with tissues and more diversified in polyploid species, to date the role ARF2 in cotton fiber cells has not been explored.

In this study, we conducted a genome-wide analysis ARF genes in three cotton species (*G. hirsutum*, *G. arboreum* and *G. raimondii*), and classified them into four clades. In *G. hirsutum* most *ARF* genes were expressed in multiple cotton tissues, among which *GhARF2b* exhibited a preferential expression in developing cotton fiber cells, and it negatively affects cotton fiber elongation but plays a role in promoting fiber initiation.

## Results

### ARF transcription factors in *G. arboreum* and *G. hirsutum*

The genome sequences of *G. raimondii* and *G. arboreum* provide us data resources to conduct a genome-wide screen of the *ARF* genes in the extent diploid progenitors of the allotetraploid *G. hirsutum*. In the previous studies, Sun et al., (2015) identified 35 *ARF* genes in *G. raimondii* [32]. To mine more ARF transcription factors in cottons the conserved domain (Pfam ID: PF06507) was used to hmmersearch against the *G. arboreum* and *G. hirsutum* genome databases, which resulted in 36 and 73 genes in *G. arboreum* and *G. hirsutum* genomes, respectively. The 36 *G. arboreum ARF* genes were designated *GaARF1*–*GaARF20*, and the 73 *G. hirsutum ARF* genes in A- and D-subgenomes were designated as *GhARF1A/D*–*GhARF21A/D* (Table 1). As those of Arabidopsis, cotton ARF proteins are composed of three domain regions, including DBD (DNA-binding Domain), MI (Middle Region) and CTD (C-terminal Domain) (Additional file 1: Figure S1).

**Table 1:** *Ka*, *Ks* and *K*a/*K*s analyses of *GhARF* genes compared with their corresponding progenitor homoeologs

Locus Name	Gene Name	Chrom	Locus Name	Gene Name	Chrom	Ka	Ks	Ka/Ks
Gh_A10G1402	GhARF1_A	A10	Cotton_A_31395	GaARF1	CA_chr1	0.0147	0.0261	0.5632
Gh_D10G0803	GhARF1_D	D10	Gorai.011G091100.1	GrARF1	Chr11	0.0019	0.0108	0.1759
Gh_A07G0411	GhARF2a_A	A07	Cotton_A_03644	GaARF2a	CA_chr1	0.0035	0.0068	0.5147
Gh_D07G0476	GhARF2a_D	D07	Gorai.001G054600.1	GrARF2a	Chr1	0.0061	0.0169	0.3609
Gh_A11G0358	GhARF2b_A	A11	Cotton_A_01955	GaARF2b	CA_chr6	0.0015	0.0151	0.0993
Gh_D11G0416	GhARF2b_D	D11	Gorai.007G044900.1	GrARF2b	Chr7	0.0028	0.0045	0.6222
Gh_D12G1909	GhARF2c_D	D12	Gorai.008G210200.1	GrARF2c	Chr8	0.0083	0.0263	0.3156
Gh_A11G1082	GhARF2d_A	A11	Cotton_A_08273	GaARF2d	CA_chr4	0.0012	0.0021	0.5714
Gh_D11G1233	GhARF2d_D	D11	Gorai.007G131900.1	GrARF2d	Chr7	0.0058	0.0181	0.3204
Gh_A08G0656	GhARF2e_A	A08	Cotton_A_22543	GaARF2e	CA_chr10	0.0032	0.0000	2.0000
Gh_D08G0758	GhARF2e_D	D08	Gorai.004G085400.1	GrARF2e	Chr4	0.0098	0.0213	0.4601
Gh_A10G0266	GhARF3a_A	A10	Cotton_A_03933	GaARF3a	CA_chr9	0.0096	0.0167	0.5749
Gh_D10G0266	GhARF3a_D	D10	Gorai.011G030900.1	GrARF3a	Chr11	0.0038	0.0125	0.3040
Gh_A06G2038	GhARF3b_A	A06	Cotton_A_40208	GaARF3b	CA_chr8	0.0019	0.0060	0.3167
Gh_D06G1415	GhARF3b_D	D06	Gorai.010G157400.1	GrARF3b	Chr10	0.0089	0.0077	1.1558
Gh_A05G1337	GhARF3c_A	A05	Cotton_A_11311	GaARF3c	CA_chr10	0.0165	0.0140	1.1786
Gh_D05G1506	GhARF3c_D	D05	Gorai.009G166100.1	GrARF3c	Chr9	0.0018	0.0076	0.2368
Gh_A09G0993	GhARF4a_A	A09	Cotton_A_01738	GaARF4a	CA_chr11	0.0017	0.0116	0.1466
Gh_A05G3908	GhARF4b_A	A05	Cotton_A_11048	GaARF4b	CA_chr10	0.0027	0.0018	1.5000
Gh_A01G0908	GhARF5a_A	A01	Cotton_A_27669	GaARF5a	CA_chr13	0.0009	0.0110	0.0818
Gh_D01G0951	GhARF5a_D	D01	Gorai.002G124400.1	GrARF5a	Chr2	0.0032	0.0094	0.3404
Gh_A05G1607	GhARF5b_A	A05	Cotton_A_16408	GaARF5b	CA_chr8	0.0046	0.0079	0.5823
Gh_D05G1792	GhARF5b_D	D05	Gorai.009G196100.1	GrARF5b	Chr9	0.0067	0.0172	0.3895
Gh_A10G0412	GhARF6a_A	A10	Cotton_A_02933	GaARF6a	CA_chr9	0.0038	0.0159	0.2390
Gh_D10G0426	GhARF6a_D	D10	Gorai.011G048200.1	GrARF6a	Chr11	0.0019	0.0047	0.4043
Gh_A05G1225	GhARF6b_A	A05	Cotton_A_26156	GaARF6b	CA_chr10	0.0034	0.0095	0.3579
Gh_D05G3848	GhARF6b_D	D05	Gorai.009G152700.1	GrARF6b	Chr9	0.0107	0.0205	0.5220
Gh_D07G1785	GhARF8a_D	D07	Gorai.001G204500.1	GrARF8a	Chr1	0.0028	0.0091	0.3077
Gh_A12G0813	GhARF8b_A	A12	Cotton_A_35443	GaARF8b	CA_chr6	0.0017	0.0054	0.3148
Gh_D12G0831	GhARF8b_D	D12	Gorai.008G097200.1	GrARF8b	Chr8	0.0039	0.0049	0.7959
Gh_A12G0483	GhARF8c_A	A12	Cotton_A_21333	GaARF8c	CA_chr6	0.0235	0.0303	0.7756
Gh_D12G0491	GhARF8c_D	D12	Gorai.008G054600.1	GrARF8c	Chr8	0.0061	0.0167	0.3653
Gh_A09G0074	GhARF8d_A	A09	Cotton_A_14740	GaARF8d	CA_chr11	0.0030	0.0131	0.2290
Gh_D09G0071	GhARF8d_D	D09	Gorai.006G008700.1	GrARF8d	Chr6	0.0031	0.0150	0.2067
Gh_A11G0231	GhARF9a_A	A11	Cotton_A_18937	GaARF9a	CA_chr10	0.0108	0.0301	0.3588
Gh_D11G0245	GhARF9a_D	D11	Gorai.007G026900.1	GrARF9a	Chr7	0.0098	0.0198	0.4949
Gh_A02G0979	GhARF9b_A	A02	Cotton_A_36154	GaARF9b	CA_chr7	0.0013	0.0044	0.2955
Gh_D03G0771	GhARF9b_D	D03	Gorai.003G078000.1	GrARF9b	Chr3	0.0019	0.0064	0.2969
Gh_A03G0274	GhARF10a_A	A03	Cotton_A_04263	GaARF10a	CA_chr7	0.0013	0.0062	0.2097
Gh_D03G1293	GhARF10a_D	D03	Gorai.003G142500.1	GrARF10a	Chr3	0.0058	0.0171	0.3392
Gh_A05G0895	GhARF10b_A	A05	Cotton_A_07064	GaARF10b	CA_chr10	0.0038	0.0102	0.3725
Gh_D05G0978	GhARF10b_D	D05	Gorai.009G107800.1	GrARF10b	Chr9	0.0025	0.0185	0.1351
Gh_A07G1254	GhARF11_A	A07	Cotton_A_31049	GaARF11	CA_chr1	0.0044	0.0105	0.4190
Gh_A10G1836	GhARF16a_A	A10	Cotton_A_23397	GaARF16a	CA_chr9	0.0019	0.0150	0.1267
Gh_D10G2093	GhARF16a_D	D10	Gorai.011G238900.1	GrARF16a	Chr11	0.0063	0.0193	0.3264
Gh_A05G3576	GhARF16b_A	A05	Cotton_A_06107	GaARF16b	CA_chr12	0.0097	0.0066	1.4697
Gh_D04G0030	GhARF16b_D	D04	Gorai.012G004800.1	GrARF16b	Chr12	0.0051	0.0087	0.5862
Gh_A09G1401	GhARF16c_A	A09	Cotton_A_24047	GaARF16c	CA_chr10	0.0031	0.0103	0.3010
Gh_D09G1405	GhARF16c_D	D09	Gorai.006G166400.1	GrARF16c	Chr6	0.0025	0.0081	0.3086
Gh_A13G2013	GhARF16d_A	A13	Cotton_A_10518	GaARF16d	CA_chr8	0.0080	0.0109	0.7339
Gh_D13G2411	GhARF16d_D	D13	Gorai.013G267100.1	GrARF16d	Chr13	0.0041	0.0223	0.1839
Gh_A05G1991	GhARF17a_A	A05	Cotton_A_16138	GaARF17a	CA_chr10	0.0030	0.0243	0.1235
Gh_D05G3805	GhARF17a_D	D05	Gorai.009G241900.1	GrARF17a	Chr9	0.0015	0.0121	0.1240
Gh_A06G0332	GhARF17b_A	A06	Cotton_A_18446	GaARF17b	CA_chr8	0.0030	0.0025	1.2000
Gh_D06G0360	GhARF17b_D	D06	Gorai.010G046000.1	GrARF17b	Chr10	0.0076	0.0099	0.7677
Gh_A11G0886	GhARF18a_A	A11	Cotton_A_14407	GaARF18a	CA_chr4	0.0041	0.0074	0.5541
Gh_D11G1034	GhARF18a_D	D11	Gorai.007G109500.1	GrARF18a	Chr7	0.0057	0.0133	0.4286
Gh_A12G1016	GhARF18b_A	A12	Cotton_A_25871	GaARF18b	CA_chr6	0.0045	0.0150	0.3000
Gh_D12G1134	GhARF18b_D	D12	Gorai.008G126200.1	GrARF18b	Chr8	0.0026	0.0201	0.1294
Gh_A06G0710	GhARF19.1a_A	A06	Cotton_A_38575	GaARF19.1a	CA_chr13	0.0048	0.0130	0.3692
Gh_D06G0818	GhARF19.1a_D	D06	Gorai.010G091300.1	GrARF19.1a	Chr10	0.0293	0.0343	0.8542
Gh_A07G2353	GhARF19.1b_A	A07	Cotton_A_05677	GaARF19.1b	CA_chr1	0.0042	0.0218	0.1927
Gh_D07G0132	GhARF19.1b_D	D07	Gorai.001G017000.1	GrARF19.1b	Chr1	0.0032	0.0113	0.2832
Gh_A05G3541	GhARF19.2_A	A05	Cotton_A_06071	GaARF19.2	CA_chr12	0.0148	0.0254	0.5827
Gh_D04G0067	GhARF19.2_D	D04	Gorai.012G009000.1	GrARF19.2	Chr12	0.0039	0.0040	0.9750
Gh_A05G0264	GhARF20_A	A05	Cotton_A_27843	GaARF20	CA_chr9	0.0020	0.0121	0.1653
Gh_D08G1407	GhARF21_D	D08	Gorai.006G045300.1	GrARF21	Chr6	0.1826	0.2293	0.7963

### Phylogenetic analysis of *Gossypium* ARF proteins

**Fig. 1:**
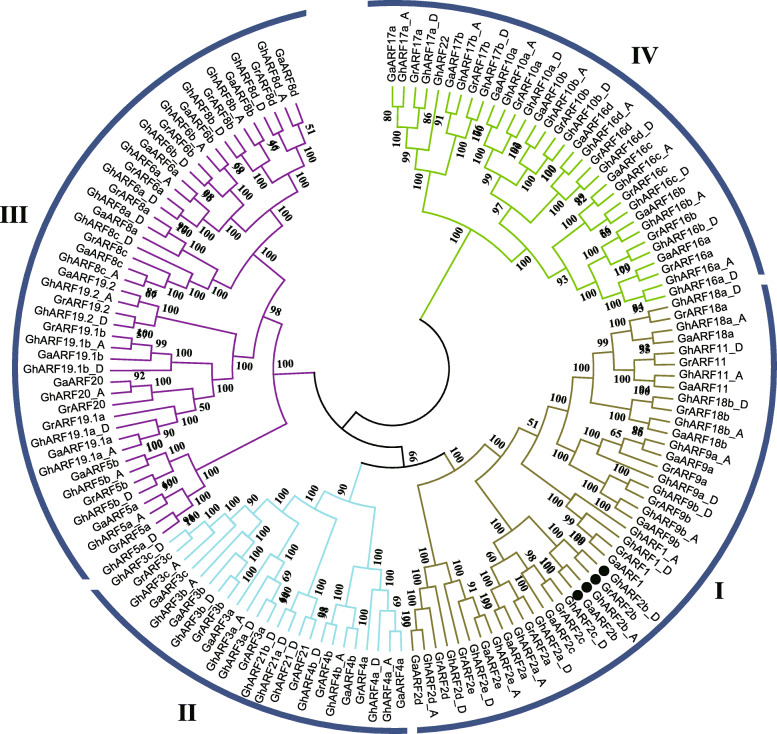
Phylogenetic trees of *Gossypium* ARFs family. 144 *Gossypium* ARFs were divided into four clades. Black dots represent the *ARF2b* genes in three *Gossypium* species

To illustrate the evolutionary relationships among the cotton ARFs, a phylogenetic tree was constructed using the protein sequences of 144 cotton ARFs, which were clustered into four clades (I–IV). The highest number of *Gossypium* ARFs are found in clade III and I, followed by clade IV and II (Fig. 1).

Overall, the expected diploid-polyploid topology is reflected in the tree for each set of orthologous/homoeologous genes, indicating general preservation during divergence of diploids and through the polyploid formation. We found that the number of ARF genes in *G. hirsutum* are approximately twice that in *G. raimondii* and *G. arboreum*, with one A_t_ or D_t_ homoeologous copy corresponding to one ortholog in each of the diploid cottons. Further, as shown in Fig. 1, the orthologous paired genes of the A genome (*G. arboreum*) and A_t_ sub-genome, or from the D genome (*G. raimondii*) and D_t_ sub-genome, tend to be clustered together and share a sister relationship.

### Divergence of ARF genes in allotetraploid *G. hirsutum* and its diploid progenitors

**Fig. 2:**
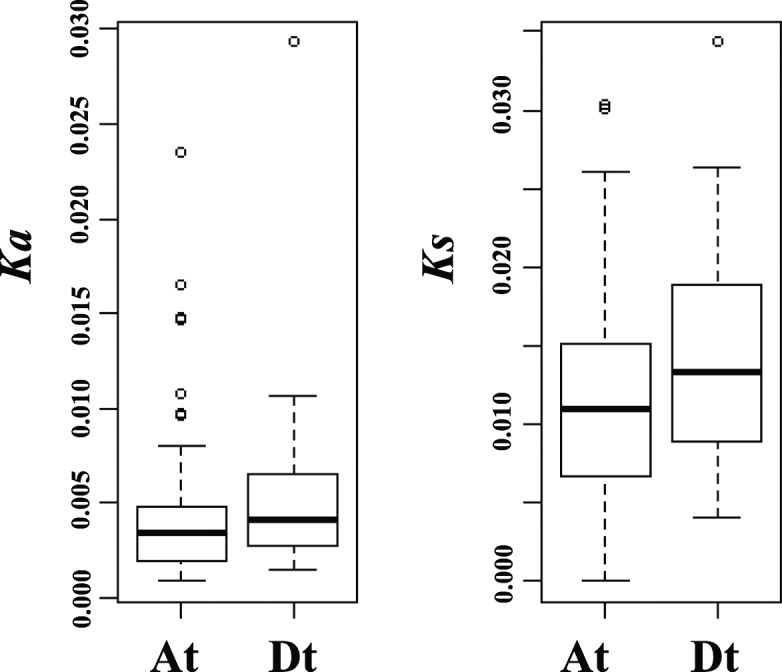
Distribution of Ka and Ks values of *ARF* genes between the A and D subgenomes versus their corresponding diploid progenitor homoeologs

The *ARF* genes in the two diploid species were then compared with *G. hirsutum* A_t_- and D_t_-subgenome homoeologs (Table 1). To explore the evolutionary relationship and possible functional divergence of *ARF* genes between the allotetraploid cotton and its extend diploid progenitors, the nonsynonymous substitution (*Ka*) and synonymous substitution values (*Ks*) and the *K*a/*K*s ratios for each pair of the genes were calculated (Table 1). By comparing the *Ka* and *Ks* values of 66 orthologous gene sets between the allotetraploid and its diploid progenitor genomes, we found that the *Ka* and *Ks* values are higher in the D_t_ subgenome than in the A_t_ subgenome (Fig. 2). These results indicate that *GhARF* genes in the D_t_ subgenome tend to have experienced faster sequence divergence than their A_t_ counterparts, suggesting an inconsistent evolution of *ARF* genes in the two subgenomes (Fig. 2).

In addition, the *Ka/Ks* ratios of one D_t_-subgenome genes (*GhARF3b_D*) and five A_t_-subgenome gene (*GhARF2e_A*, *GhARF3c_A*, *GhARF4b_A*, *GhARF16b_A* and *GhARF17b_A*) are greater than 1 (Table 1), suggesting that these genes have under positive selections after divergence of *G. hirsutum* from diploid ancestors, and may have gained new functions.

### Expression analysis of *GhARF* genes in different cotton tissues

The expression profile of a gene family can provide valuable clues to possible functions of each genes. Analysis of 73 *GhARF* genes showed that most genes have different spatial expression patterns. For instance, *GhARF1, GhARF2a, GhARF2b* and *GhARF2c* were expressed in all the tissues of cotton examined (Additional file 2: Figure S2), whereas *GhARF3a* and *GhARF3c* were expressed preferentially in the pistils and ovules. Compared to *GhARF5b*, *GhARF5a* showed higher expressions in the root, pistil and ovule organs. Transcripts of *GhARF3c* and *GhARF4a*, *GhARF9a* and *GhARF9b* were most abundant in stem and root, respectively. Over half of *GhARF* genes showed a relatively high level of transcript accumulation in leaf. Notably, there are more than 10 genes (including *GhARF1*, *GhARF2a*, *GhARF2b*, *GhARF8a*, *GhARF9a*, *GhARF10b*, *GhARF11*, *GhARF16a*, *GhARF18* and *GhARF19*) that were highly expressed in cotton fiber cells at the fast elongation stage (5 dpa).

Among them, *GhARF2* genes showed the highest expression in fiber (5 dpa) and were located in the Clade I of phylogenetic tree (Fig. 1), suggesting that they may function in cotton fiber development. Previous studies have demonstrated that ARF2 plays a role in transcriptional regulation in auxin-mediated cell division [30], leaf longevity [33], response to stress [34], regulation of fruit ripening [31] and so on. As GhARF2s shown pleiotropic effects on plant development [35], we decided to identify the major GhARF2s in regulation of cotton fiber elongation in subsequent experiments.

### *GhARF2* had a high expression pattern during fiber elongation process

**Fig. 3:**
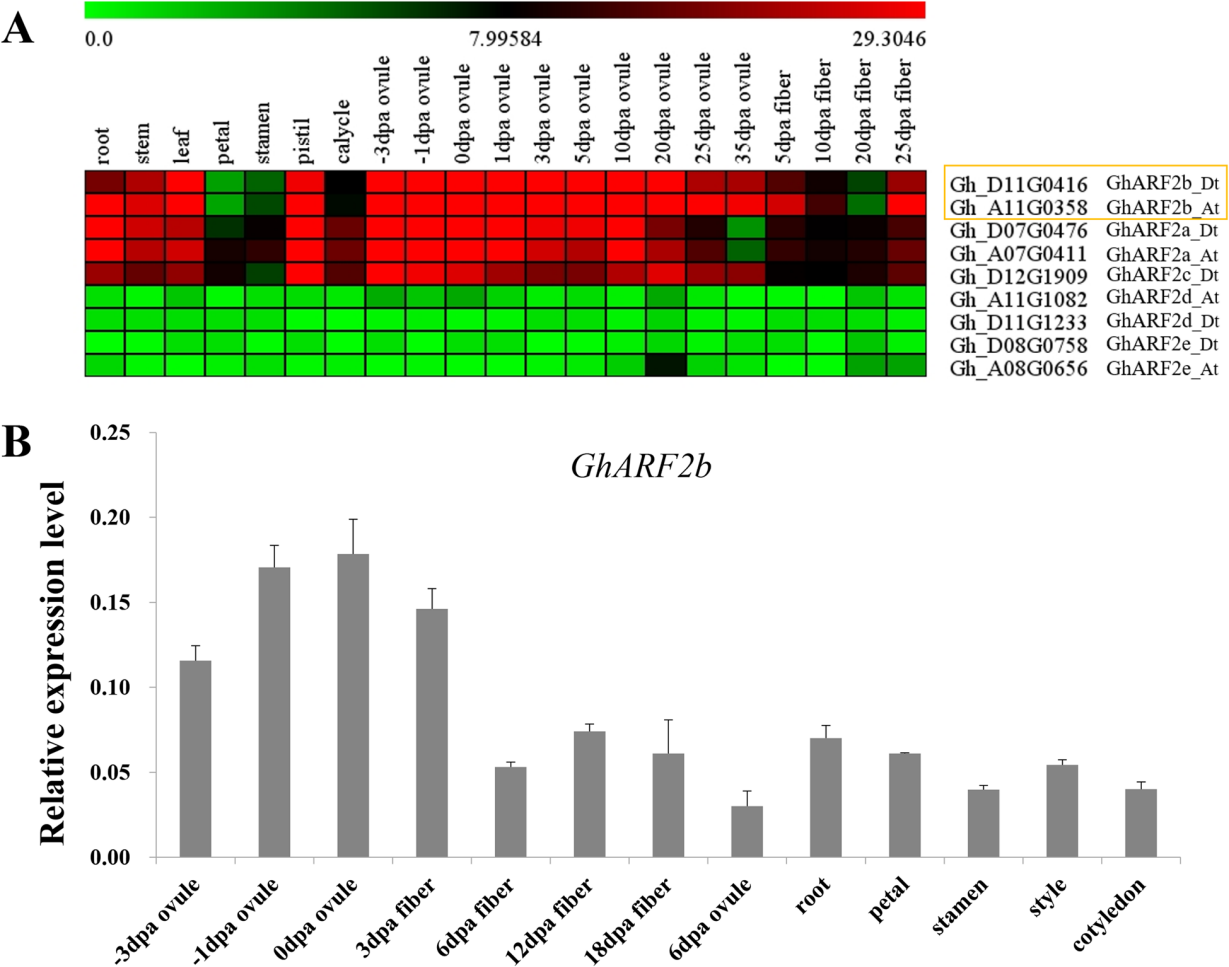
Expression patterns of *GhARF2* in different cotton tissues and fiber cells of different stages. **a** Expression profiles of nine *GhARF2* genes based on the RPKM values of RNA-seq data. *GhARF2b* was highlighted in yellow box. **b** qRT-PCR analyses of *GhARF2b* expression across different cotton tissues. The expression is relative to *GhHIS3.* Error bar indicates stdev.s level of three qRT-PCR assays

There are nine *ARF2* genes in *G. hirsutum* (*GhARF2c_At* not annotated), we first examined their expression profiles in different tissues in cotton (Fig. 3). Based on the RNA-seq data (Zhang et al., 2015), *GhARF2a*, *GhARF2b* and *GhARF2c* genes had higher expression levels in various tissues than *GhARF2d* or *GhARF2e* (Fig. 3a). Among them, in 5 dpa fiber, the expressions of *GhARF2b* were 1.1–37 folds to other four *GhARF2* genes. Whereas in ovule (0dpa), *GhARF2b* showed 1.2–15 folds higher expressions than others. Thus, the transcripts of *GhARF2b* homoeologs (*GhARF2b_At and GhARF2b_Dt*) were enriched and abundant in cotton fiber and ovule cells (Fig. 3a). Subsequent quantitative RT-PCR (qRT-PCR) confirmed the expression pattern, and *GhARF2b* showed 3.6–9 folds higher expressions in fiber (3dpa) or ovule (0dpa) than other tissues (Fig. 3b). The highly up-regulated expression in fiber cell suggested that GhARF2b has been recruited to act primarily in cotton fiber.

### *GhARF2b* overexpression represses cotton fiber elongation

**Fig. 4:**
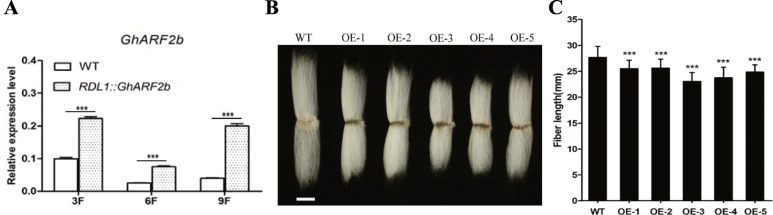
GhARF2b affects fiber length and fiber related gene expression in *RDL1::GhARF2b* transgenic cotton. **a** Expression of *GhARF2b* in 3, 6 and 9 dpa fibers in the *RDL1::GhARF2b* overexpression (OE) lines compared to wild-type (WT). **b** Fiber phenotype of *RDL1::GhARF2b* and wild-type (WT) cotton cultivated in farm on shanghai, bar = 10 mm. **c** Statistical analysis of *RDL1::GhARF2b* and wild-type (WT) mature fiber length. Error bar indicates standard deviation; *** denotes significant difference from wild type (Student’s t-test, *P* < 0.001, *n* = 30)

To test the function of *GhARF2b*, we constructed the vectors to over-express and down-regulate *GhARF2b_Dt* in *G. hirsutum* by using the fiber-specific *GhRDL1* promoter [8, 19, 36]. The expression levels of *GhARF2b* in transgenic cotton were clearly elevated in the overexpression lines according to qRT-PCR analysis; for example, the *GhARF2b* transcript abundance was about two-fold higher in the OE-3 than in the wild-type cotton fiber cells (Fig. 4a). However, *GhARF2b* did not stimulate fiber cell elongation, rather, it resulted in shorter fiber (Fig. 4b, c).

**Fig. 5:**
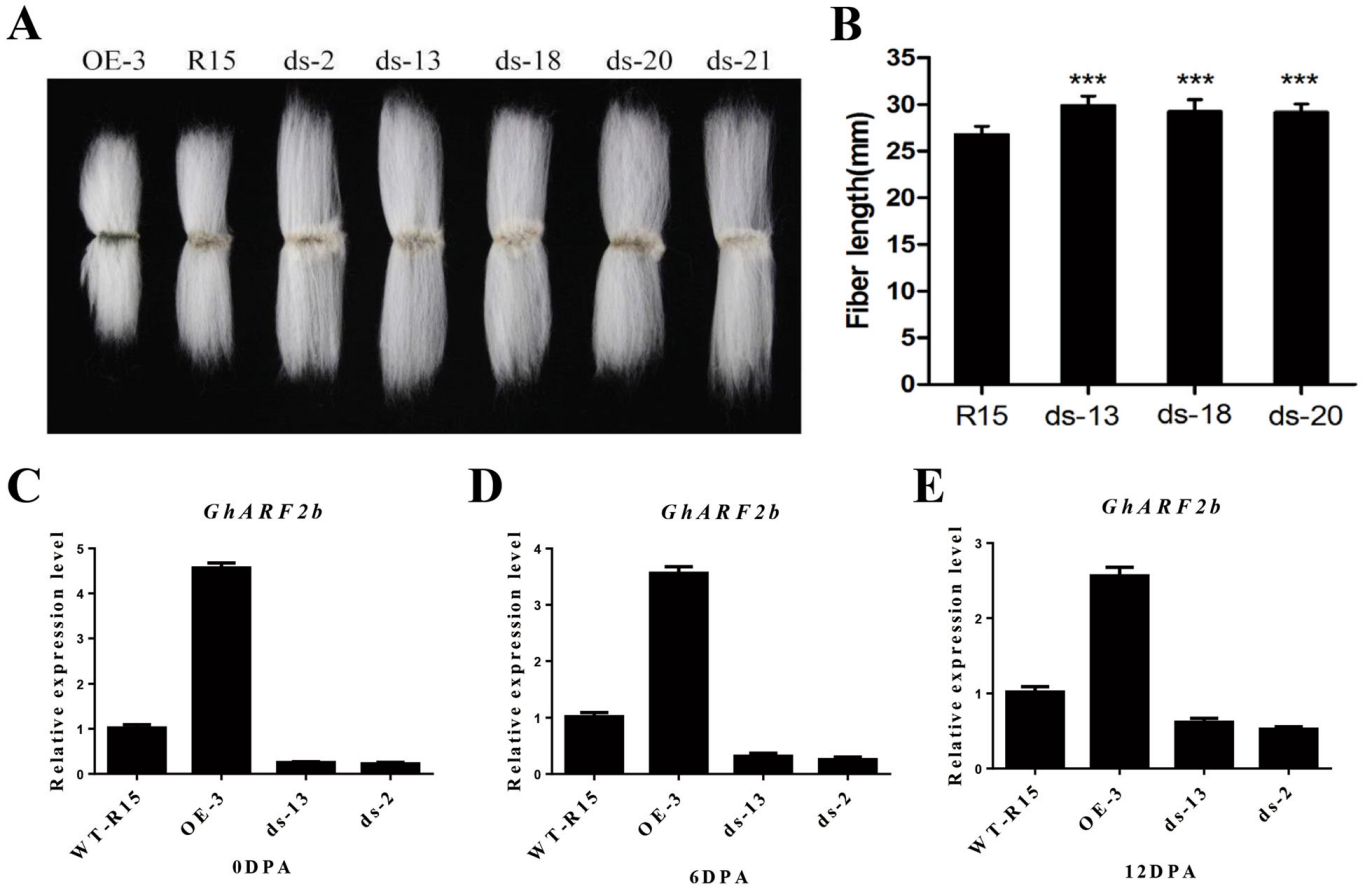
GhARF2b affects fiber length and gene expression in *RDL1::GhARF2b* RNAi transgenic cotton. **a** Fiber phenotype of *RDL1::GhARF2b* (OE), *RDL1::GhARF2b* RNAi (ds) and wild-type (R15) cotton cultivated in farm on shanghai, bar = 10 mm. **b** Statistical analysis of *RDL1::GhARF2b* RNAi (ds) lines and wild-type (WT) mature fiber length. Error bar indicates standard deviation; *** denotes significant difference from wild type (Student’s t-test, *P* < 0.001, *n* = 30). **c** Expression of *GhARF2b* in 0 dpa ovules in the overexpression (OE), RNAi (ds) lines compared to wild-type (WT). **d** Expression of *GhARF2b* in 6-dpa fibers in the overexpression (OE), RNAi (ds) lines compared to wild-type (WT). **e** Expression of *GhARF2b* in 12-dpa fibers in the overexpression (OE) and the RNAi (ds) lines compared to the wild-type (WT). Error bar indicates stdev.s level of three qRT-PCR assays

On the contrary, suppressing *GhARF2b* expression by RNAi resulted in longer fibers (Fig. 5a, b). The expression levels of *GhARF2b* in RNAi cottons in the RNAi lines were about 3 ~ 5-fold down-regulated in cotton fiber of 0DPA, 6DPA and 12DPA (Fig. 5c-e). Together, these data suggest that GhARF2b acted as a negative regulator of fiber cell elongation, at least when its expression exceeded the threshold. Alternatively, it may function in other aspects of cotton fiber development.

### GhARF2b interacted with GhHOX3

**Fig. 6:**
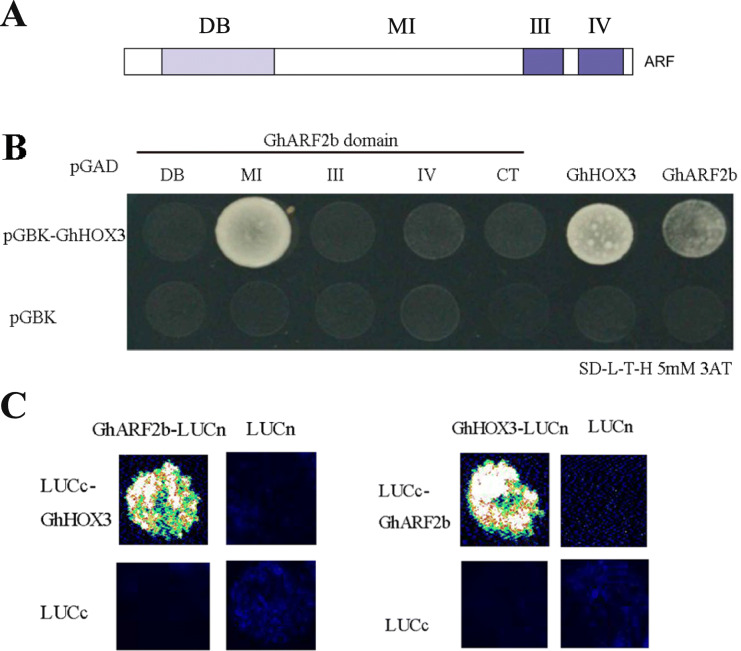
Interaction of GhARF2b and GhHOX3. **a** Diagram of protein domains in ARF protein. **b** GhARF2b and GhARF2b middle region can interact with GhHOX3 in Y2H assay. The figure shows yeast grown on SD/−Leu-Trp-His with 5 mM 3AT (3-amino-1,2,4-triazole). **c** GhHOX3 and GhARF2b are interchangeably fused to the carboxyl- and amino-terminal of firefly luciferase (LUC, LUCc and LUCn), transiently co-expressed, and LUCc or LUCn was co-expressed with each other or with each unfused target protein as the control. Fluorescence signal intensities represent their binding activities

The homeodomain-leucine zipper (HD-ZIP) transcription factor, GhHOX3, plays a determinant role in controlling cotton fiber elongation [5]. We used the yeast two-hybrid system (Y2H) to screen a cotton fiber cDNA library for GhHOX3 interacting proteins. GhARF2 was among the top five interacting factors of the target proteins. In further yeast two-hybrid assays, GhARF2b and GhARF2b middle region strongly interacted with GhHOX3 (Fig. 6a, b). We also used bimolecular fluorescence complementation (BiFC) assays to confirm the interaction between GhARF2b and GhHOX3 (Fig. 6c).

### The transcriptional activities of GhHOX3 target genes were repressed by GhARF2b protein interactions

**Fig. 7:**
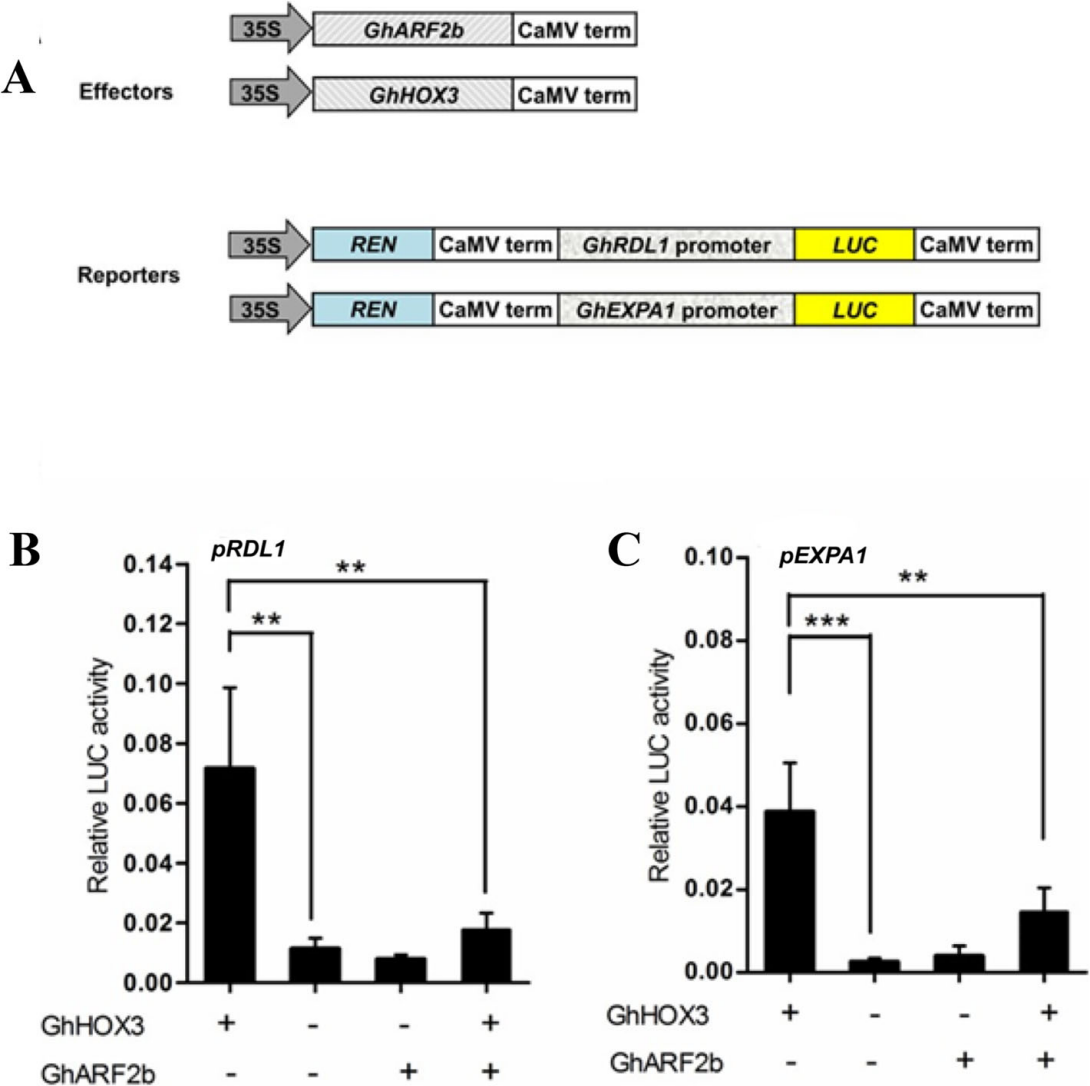
GhARF2b represses the function of GhHOX3. **a** Diagram of vectors used in this assay. **b** GhARF2b affects the activation of GhHOX3 to *GhRDL1*. **c** GhARF2b affects the activation of GhHOX3 to *GhEXPA1*. Data are presented as mean ± S.D., *n* = 3, **P < 0.01, ***P < 0.001, Student’s t-test)

Given the fact that GhARF2b represses cotton fiber elongation, we tested the two protein interactions would affect the transcriptional activation of GhHOX3 target genes. Two cell wall protein coding genes [19, 36], *GhRDL1* and *GhEXPA1*, are direct targets of GhHOX3 in promoting the fiber elongation [5]. We used a dual-luciferase assay system to study the effect of GhARF2b on activity of GhHOX3 protein (Fig. 7a). The level of the luciferase activity driven by GhRDL1 and *GhEXPA1* promoters was significantly increased when GhHOX3 was expressed (Fig. 7b, c). In contrast, activation of GhHOX3 to *GhRDL1* or *GhEXPA1* promoters was significantly repressed by GhARF2b (Fig. 7b, c). These results further supported that interaction of GhARF2b with GhHOX3 results in a much lower activity of targets gene activation, thus cotton fiber elongation was disturbed.

### *GhARF2b* overexpression enhances cotton fiber initiation

**Fig. 8:**
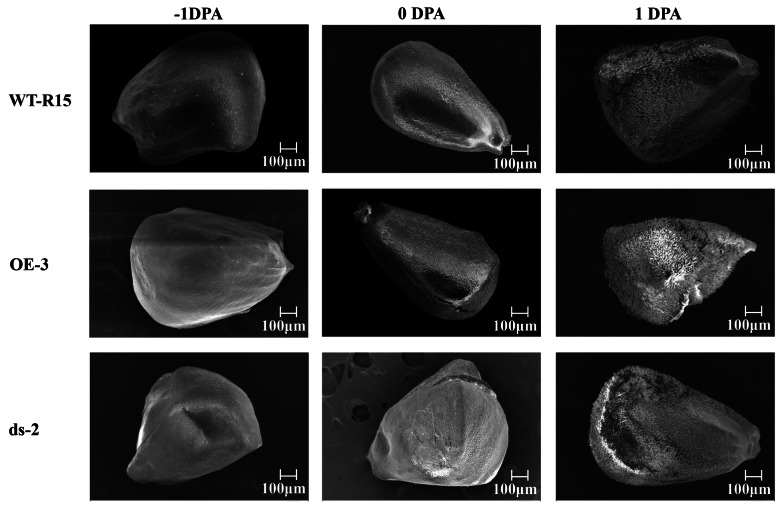
SEM images of WT-R15, OE-3 (over-expression line) and ds-2 (RNAi line) ovules at −1, 0 and 1 DPA. Bar = 100 μm [60 × magnification]

**Fig. 9:**
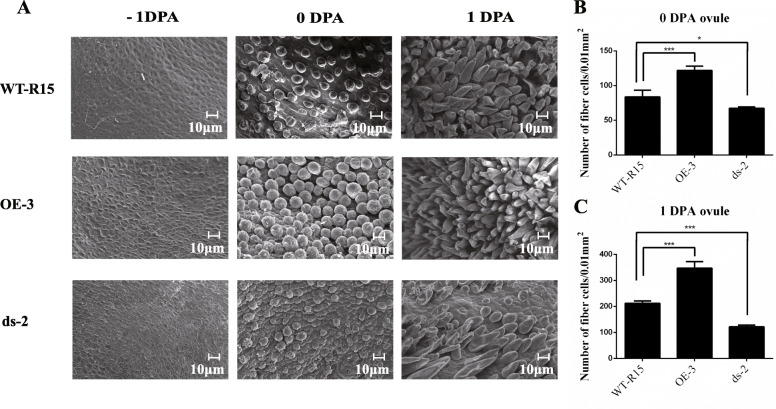
GhARF2b enhances the fiber initiation. **a** SEM images of WT-R15, OE-3 (over-expression line) and ds-2 (RNAi line) ovules at − 1, 0, 1 DPA. Bar = 100 μm [700 × magnification for ovules of − 1 and 0 DPA, 500 × magnification for 1-DPA ovules]. **b** Number of fiber cells per 0.01 mm^2^ of 0-DPA ovules in the overexpression (OE-3) and RNAi (ds-2) cotton lines compared to wild-type (WT-R15). Error bar indicates standard deviation; *** denotes significant difference from wild-type (Student’s t-test, *P* < 0.001, *n* = 30); * denotes significant difference from wild type (Student’s t-test, *P* < 0.05, *n* = 30). **c** Number of fiber cells per 0.01 mm^2^ of 1-DPA ovules in the overexpression (OE-3) and RNAi (ds-2) cotton lines compared to wild-type (WT-R15). Error bar indicates standard deviation; *** denotes significant difference from wild type (Student’s t-test, *P* < 0.001, *n* = 30)

Next, we examined the effects of *GhARF2b* up-regulation on cotton fiber initiation. The over-expression line OE-3 and RNAi line ds-2 were selected for analyses. The SEM with 60 × magnification of ovules of WT-R15, OE-3 and ds-2 collected at − 1, 0, 1 DPA were observed (Fig. 8). The cotton fiber initiation of the − 1-DPA ovules did not present differences among the three types of cottons, however, the 0- and 1-DPA ovules of OE-3 and ds-2 lines showed higher and lower densities of fiber initials compared to the wild-type control (Fig. 8). Further, we magnified the SEM views of ovules to 500–700× (Fig. 9). Obviously, at the fiber initiation stage (0, 1 DPA), the fiber initial density of the OE-3 was increased by about 1.5-fold compared with that of the wild-type, in contrast, the fiber initial density of the ds-2 line was reduced (Fig. 9a-c). These results support a role of GhARF2b in promoting cotton fiber cell initiation.

## Discussion

Currently, more than 20 cotton genome sequences have been assembled and released, including diploid *G. raimondii* [37, 38], *G. herbaceum* and *G. arboreum* [39–41] and tetraploid *G. hirsutum, G. barbadense, G. tomentosum, G. mustelinum* and *G. darwinii* [41–49]. These genome sequences provided a platform for dissecting gene functions by forward and reverse genetics and would accelerate the rate of molecular breeding in cotton. Here, based on these high-quality genome sequences, we additionally characterized 36 ARF genes in *G. arboreum* and 73 in *G. hirsutum*, adding valuable data to understanding the distribution and evolution of ARF genes in cotton plants.

After whole genome duplication, the amplified genes generally undergo the events of functional loss, or neofunctionalization or subfunctionalization [50]. In this study, we found that six *GhARF* genes (five from A_t_ subgenome) have experienced relatively faster positive selection compared to its diploid progenitors. Thus, duplicated genes from A_t_ and D_t_ subgenomes might be functionally diverged in the allotetraploid cotton after the merge of the two genomes. In addition, the *GhARF* genes expression profiles analyzed from the RNA-seq data showed subgenome-biased expression that might undergone functional divergence during the evolution. For instance, unequal expressions were observed in A and D-subgenome genes, including *GhARF3c*, *GhARF16c*, *GhARF18a* and *GhARF20*. These massive alterations in gene expression can cause distinct function and may just be one of the important features emerging from polyploid [8, 51]. During the evolution of allopolyploid, some duplicate gene pairs (homoeologs) are expressed unequally, as also proved in the allopolyploid cotton genome with the features of asymmetrical evolution [42]. The above results indicated that this suite of unequally expressed genes may be a fundamental feature of allopolyploids.

Previous studies showed that ARF family genes have been identified in many plant species, including 23 ARF genes in *Arabidopsis thaliana* [26], 25 in *Oryza sativa* [27], 39 in *Populus trichocarpa* [52], 31 in *Zea mays* [53], 15 in *Cucumis sativus* [54] and 35 in *G. raimondii* [32]. Auxin response factors (ARFs) are important in plant development as they play crucial roles in regulating a variety of signaling pathways [24, 25]. According to their functions, ARF proteins are divided into two classes: transcriptional activators and transcriptional repressors [24]. Many studies have revealed their regulatory roles in regulating various aspects of cellular activities [35, 55–57]. As transcriptional repressors, ARF2 was involved in the regulation of K^+^ uptake by repressing *HAK5* transcription in Arabidopsis [34]. In addition, ARF2 is regulated by a variety of upstream factors at the transcription and protein levels, and participated in the pathways of auxin, gibberellin, oleoresin, ethylene and abscisic acid [28, 29, 31, 58].

In cotton, Zhang et al. uncovered that expression of the IAA biosynthetic gene, *iaaM*, can significantly increase IAA levels in the epidermis of cotton ovules at the fiber initiation stage, and increased the number of lint fibers and lint percentage in a 4-year field trial. They proved that the lint percentage of the transgenic cotton was increased in transgenic plants with a 15% increase in lint yield [59]. Han et al. found that the auxin response factor gene (*GhARF3*) was highly correlated with fibre quality by using the haplotype analysis and transcriptomic data. Above all, auxin signaling plays an essential role in regulating fibre development. In addition, Xiao et al. showed that *G. hirsutum ARF* genes promoted the trichome initiation in transgenic Arabidopsis plants [60]. They identified 56 *GhARF* genes in their study, including three GhARF2 genes [60]. They showed that *GhARF2–1* could be exclusively expressed in trichomes, and overexpression of *GhARF2–1* in Arabidopsis can enhance trichome initiation. But their study did not perform the cotton transformation to test the function of *GhARF2–1* in cotton fiber cell.

## Conclusions

In our study, we reported 73 *GhARF* genes in *Gossypium hirsutum* genome, including 9 *GhARF2* genes. Among them, GhARF2b, was specifically higher expressed in developing fibers. Overexpression of GhARF2b represses fiber elongation, and RNAi silencing of GhARF2b promotes the fiber longer. Through yeast two-hybrid assays and the Dual-LUC experiment, GhARF2b plays a negative role in controlling cotton fiber elongation by interacting with GhHOX3. Further, GhARF2b was shown to promote the production of fiber initials, suggesting that auxin is an important player in controlling cotton fiber development. The auxin signaling pathways in developing cotton fiber cells deserve further investigation.

## Methods

### Identification of *Gossypium* species ARF factors

*G. raimondii* [37], *G. arboreum* [39], *G. hirsutum* [42] genome sequences were acquired from the CottonGen database [61]. We developed a Hidden Markov Model [62] profile matrix of ARF factors (Pfam ID: PF06507) via the hmmbuild program [63] with default parameters to identify *Gossypium* ARF transcription factor proteins. SMART conserved domain search tool [64] and Pfam databases [65] were used to identify the conserved domain.

### Sequence alignment, Ka, Ks analyses and phylogenetic analyses

*Gossypium* ARF factor amino acids and nucleotide sequences were aligned by MAFFT software with the G-INS-i algorithm [66]. Ka, Ks and Ka/Ks values for each gene pairs between diploid and allotetraploid were calculated by DnaSP v5 [67]. The Neighbor-Joining (NJ) phylogenetic tree was drawn by MEGA 5.03 [68] by sampling 1000 bootstrap replicates based on the ARF whole protein sequences.

### Gene expression analyses based on transcriptome

Raw RNA-Seq data were downloaded from the NCBI Sequence Read Archive (https://www.ncbi.nlm.nih.gov/bioproject/PRJNA248163) [42], including *G. hirsutum* seed, root, stem, leaf, torus, petal, stamen, ovary, calyx, ovule (− 3 dpa, − 1 dpa, 0 dpa, 1 dpa, 3 dpa, 5 dpa, 10 dpa, 20 dpa, 25dpa, 35dpa) and fiber (5 dpa, 10 dpa, 20 dpa, 25dpa). The method of gene expression analyses based on transcriptome was same to our previous study [69]. Differentially expressed genes were determined based on the following criteria: more than two-fold change and *p*-value less than 0.05. Multiple Experiment Viewer (MeV) [70] was used to display the gene expression values.

### Plant materials and growth conditions

*Gossypium hirsutum* cv. R15 wild type plants were obtained from Institute of Cotton Research, Shanxi Academy of Agricultural Sciences, Yuncheng, Shanxi, China. Upland cotton R15 plants and its transgenic lines were grown in a greenhouse or in a field under standard farming conditions, which is in the experimental field of Chinese Academy of Sciences in Shanghai according to relevant national approvals for biotechnology research (China, http://pg.natesc.gov.cn/sites/pg/). The greenhouse is in a controlled environment at 28 °C day/20 °C night, a 16-h light/8-h dark photoperiod. Cotton tissues, including roots, cotyledon, petal, stamen, style, ovules (− 3, − 1, 0 and 6 dpa) and fiber (3, 6, 12 and 18 dpa) were collected for expression analyses. Fibers were collected by scraping the ovule in liquid nitrogen. All these tissues were frozen in liquid nitrogen immediately after sampling and stored at − 80 °C until RNA extraction. Three times were repeated for all these treatments.

### qRT-PCR analyses

All cotton samples were ground in liquid nitrogen and total RNAs of these cotton tissues were extracted using the RNAprep pure plant kit (TIANGEN, Shanghai, China) following the manufacturer’s protocol. The method of qRT-PCR analyses was same to our previous study [69]. The forward and reverse primers of specific gene for quantitative real-time PCR (qRT-PCR) analyses, were designed using the Primer5 software (Additional file 3: Table S1). Analyses were performed with SYBR-Green PCR Mastermix (TaKaRa) on a cycler (Mastercycler RealPlex; Eppendorf Ltd., Shanghai, China). The internal gene was *G. hirsutum histone-3* (*GhHIS3*, AF024716), and the 2-∆∆Ct method was used to calculate the relative amount of amplified product [71]. Relative expression levels among different organs of *G. hirsutum* samples were normalized by calibrating with the WT samples.

### Cotton transformation and fiber length analysis

The open reading frame (ORF) of *GhARF2b* was PCR-amplified from a *G. hirsutum* cv R15 fiber cDNA library with PrimeSTAR HS DNA polymerase (Takara Biomedical Technology Co. Ltd., Beijing, China) and inserted into the *pCAMBIA2301* vector to construct *RDL1::GhARF2b*. For *35S::dsGhARF2b*, sense and antisense: *GhARF2b* fragments, separated by a 120-bp intron of the *RTM1* gene from *A. thaliana*, were cloned into *pCAMBIA2301*. Primers used in this investigation are listed in Additional file 3: Table S1. The binary constructs were transferred into *Agrobacterium tumefaciens*. Cotton transformation was conducted as reported in Shangguan et al. [72]. Transgenic cotton plants were grown in glasshouse or field. β-glucuronidase (GUS) staining and PCR amplification were performed to identify the transgenic lines of T_0_ and subsequent generations. Thirty seeds from each plant were harvested to statistics fiber length.

### Yeast two-hybrid assay

Yeast two-hybrid analysis were carried out using the Matchmaker GAL4 Two-Hybrid System as performed previously [5]. Briefly, for the yeast two-hybrid assays, the full-length ORF of GhHOX3 inserted into pGBKT7 (Clontech) and GhARF2b or GhARF2b different domains into pGADT7 (Clontech). Plasmids were co-transferred into yeast strain AH109 by the LiCl-PEG method, and SD/−Leu/−Trp/−His selective plates containing 5 mM 3-AT (3-amino-1,2,4,-triazole) were used to detect the protein-protein interactions. pGADT7 and pGBKT7 empty vectors were used as controls. Three biological duplications for each transformation were performed.

### BiFC and dual-luciferase (dual-LUC) assays

We performed the BiFC assays following previous reports [73, 74]. In summary, CDSs of GhARF2b and GhHOX3 were amplified and cloned into JW771 and JW772 vectors, respectively. Each gene was fused to the carboxyl-terminal half (cLUC-GhARF2b/GhHOX3) and the amino-terminal half (GhARF2b/GhHOX3-nLUC) of luciferase (LUC), respectively. cLUC and nLUC were used as controls. Assays were finished as described [5, 75].

The Dual-LUC assay was performed as reported [5, 76]. Briefly, the promoters containing intact L1-boxes of *GhRDL1* and *GhEXPA1* were inserted into pGreen-LUC vector with a firefly LUC reporter gene. Then, the constructs were transferred into *Agrobacterium tumefaciens* cell with a co-suppression repressor plasmid pSoup-P19. Transient transformation was conducted by infiltrating the *A. tumefaciens* cells into *N. benthamiana* leaves. The total protein was extracted from the infected area after 3 days. The Dual-Luciferase Reporter Assay System (Promega) was used to detect the fluorescent values of LUC and REN with a luminometer (BG-1, GEM Biomedical Inc.). The value of LUC was normalized to that of REN. Three biological replicates were measured for each experiment.

### Microscope observation

Images were generated with an optical microscope (BX51, Olympus). For scanning electron microscope images, cotton ovules (− 1, 0, 1DPA) were attached with colloidal graphite to a copper stub, frozen under vacuum and visualized with a scanning electron microscope (JSM-6360LV, JEOL).

## Supplementary Information

**Additional file 1: Figure S1.** Multiple alignment of GrARF2 (*Gossypium raimondii* ARF2) and AtARF2 protein sequences.

**Additional file 2: Figure S2.** Expression patterns of *ARF* genes in *G. hirsutum* based on RNA-seq data. FPKM represents fragments per kilobase of exon model per million mapped reads. DPA, days post-anthesis.s.

**Additional file 3: Table S1.** List of forward and reverse primers used for this study.

## Data Availability

The genome sequences of three cotton species and the genome annotation gff3 file were downloaded from the CottonGen database (https://www.cottongen.org/data/download) [59]. Raw RNA-Seq data for *G. hirsutum* seed, root, stem, leaf, torus, petal, stamen, ovary, calyx, ovule and fiber were downloaded from the NCBI Sequence Read Archive (https://www.ncbi.nlm.nih.gov/bioproject/PRJNA248163) (NCBI Sequence Read Archive SRR1695173, SRR1695174, SRR1695175, SRR1695177, SRR1695178, SRR1695179, SRR1695181, SRR1695182, SRR1695183, SRR1695184, SRR1695185, SRR1695191, SRR1695192, SRR1695193,SRR1695194, SRR1768504, SRR1768505, SRR1768506, SRR1768507, SRR1768508, SRR1768509, SRR1768510, SRR1768511, SRR1768512, SRR1768513, SRR1768514, SRR1768515, SRR1768516, SRR1768517, SRR1768518 and SRR1768519) [30]. The *G. hirsutum histone-3* (*GhHIS3*, AF024716) gene was downloaded from the National Center for Biotechnology Information (NCBI) database, which were used as internal references. The conserved domain of ARF transcription factors (Pfam ID: PF06507) was downloaded from the Pfam databases (http://pfam.xfam.org/family/PF06507#tabview=tab3). All other data generated or analyzed during this study are included in this published article and its Additional files.

## Abbreviations

ARFs: Auxin response factors
Dpa: Days post anthesis
FPKM: Fragments per kilobase of transcript per million mapped fragments
*G. arboreum*: *Gossypium arboreum*
*G. hirsutum*: *Gossypium hirsutum*
*G. raimondii*: *Gossypium raimondii*
WT: wild type
OE: Overexpression
ds: RNAi
qRT-PCR: Quantitative real-time polymerase chain reaction
