# Supplementary figures and images for: Characterization of cotton ARF factors and the role of GhARF2b in fiber development
Source: BMC Genomics. 2021 Mar 22;22:202. doi: 10.1186/s12864-021-07504-6 (PMC7986310; doi:10.1186/s12864-021-07504-6)

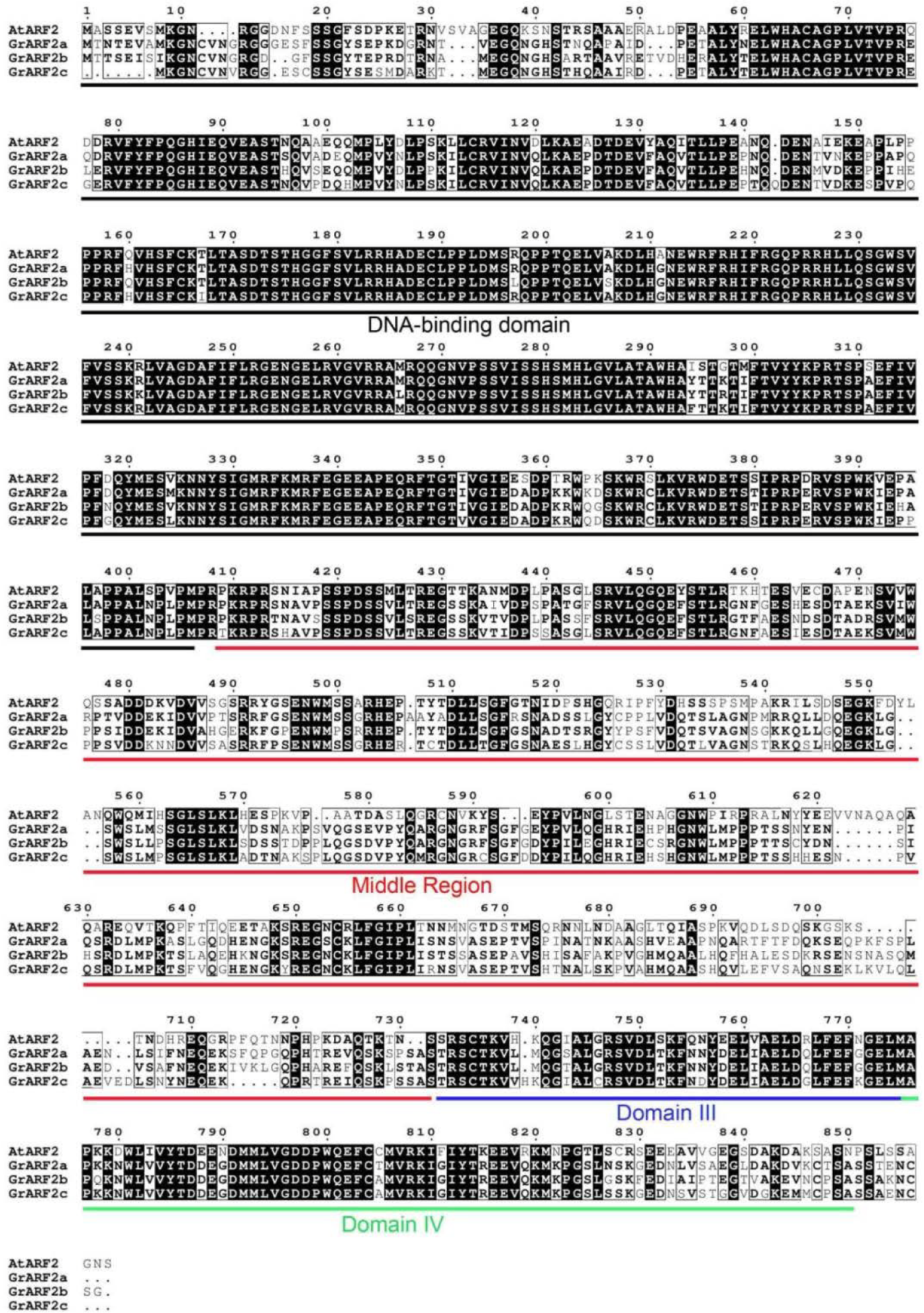

Supplement: Supplementary file 1 — Additional file 1: Figure S1. Multiple alignment of GrARF2 (Gossypium raimondii ARF2) and AtARF2 protein sequences. [file 12864_2021_7504_MOESM1_ESM.tif]

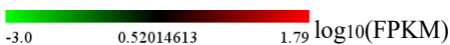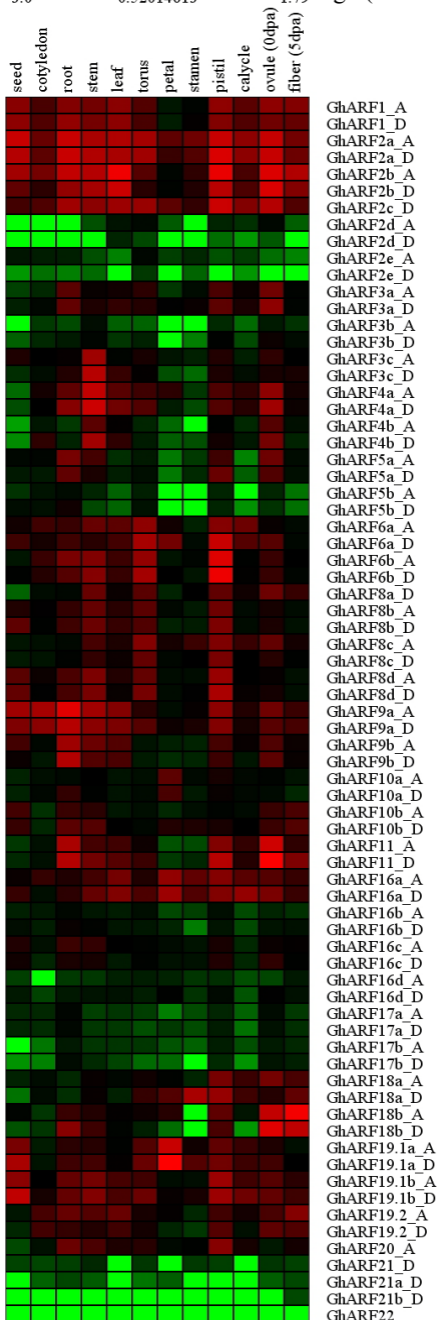

Supplement: Supplementary file 2 — Additional file 2: Figure S2. Expression patterns of ARF genes in G. hirsutum based on RNA-seq data. FPKM represents fragments per kilobase of exon model per million mapped reads. DPA, days post-anthesis.s. [file 12864_2021_7504_MOESM2_ESM.pdf]
