# Supplementary material for: Characterization of cotton ARF factors and the role of GhARF2b in fiber development
Source: BMC Genomics. 2021 Mar 22;22:202. doi: 10.1186/s12864-021-07504-6 (PMC7986310; doi:10.1186/s12864-021-07504-6)
Supplement: Supplementary file 3 — Additional file 3: Table S1. List of forward and reverse primers used for this study. [file 12864_2021_7504_MOESM3_ESM.docx]

**TABLE S1.** List of forward and reverse primers used for this study.

| Primer | Sequence (5'-3') | Purpose |
| --- | --- | --- |
| Kan-F | GGCGATACCGTAAAGCACGAGGAA | Transgenic identification |
| Kan-R | GCTATGACTGGGCACAACAGACAAT | Transgenic identification |
| GhARF2b672-AS | AGGCCAGTGAATTCCACCCGATGGGCGTTGACAATAGAACAGC | Transgenic identification |
| GhARF2b603-AS | GGATCCCGTATCGATGCCCACCCTTACTCAATAGATCCCAGCTATGT | Transgenic identification |
| GhARF2b-RT-F | CCAGAAGGAATCCACTTTGC | qRT-PCR |
| GhARF2b-RT-R | CCAGCTCACATCAGCATACG | qRT-PCR |
| ubiqutin-RT-F | CAGCAGGGTCGCTTGTAGTGT | qRT-PCR |
| ubiqutin-RT-R | CGGTCAGTTTAGCCGTTGGTA | qRT-PCR |
